# Dysfunction of natural killer cells promotes immune escape and disease progression in endometriosis

**DOI:** 10.3389/fimmu.2025.1657605

**Published:** 2025-09-05

**Authors:** Weiyu Jiang, Wen Xu, Feng Chen

**Affiliations:** ^1^ Department of Obstetrics, Obstetrics and Gynecology Center, The First Hospital of Jilin University, Changchun, Jilin, China; ^2^ Department of General Gynecology I, Obstetrics and Gynecology Center, The First Hospital of Jilin University, Changchun, Jilin, China

**Keywords:** endometriosis, natural killer cells, immune surveillance, cytotoxicity, cytokines, immunotherapy

## Abstract

Endometriosis (EMs) is a chronic inflammatory disorder characterized by dysregulated innate immunity, particularly impaired cytotoxic function of natural killer (NK) cells. As pivotal effectors of the innate immune response, NK cells fail to eliminate ectopic endometrial lesions due to aberrant receptor–ligand interactions, elevated levels of immunosuppressive cytokines (TGF-β, IL-6, and IL-10), and dysfunction of adhesion molecules. This compromised immune surveillance facilitates the survival and implantation of ectopic lesions, contributing to the hallmark symptoms of pain and infertility. Recent immunotherapeutic strategies, including NK cell checkpoint blockade (anti-NKG2A, anti-PD-1), IL-2-based activation, and adoptive NK cell transfer—seek to restore NK cell cytotoxicity and reestablish immune homeostasis. This review summarizes current advances in understanding NK cell dysfunction in EMs, emphasizing its central role in immune evasion and the therapeutic promise of targeting innate immune pathways.

## 1 Introduction

Endometriosis (EMs) is a chronic gynecological disorder characterized by low cure rates and high recurrence, affecting approximately 10% to 15% of women of reproductive age (1). Epidemiological studies indicate that up to 70% of affected individuals experience chronic pelvic pain (2), while approximately 50% suffer from infertility, substantially compromising the health and quality of life of women in their reproductive years (3). Although the retrograde menstruation theory remains the most widely accepted etiology of EMs, additional contributing factors, including genetic predisposition, immune dysfunction, and chronic inflammation, have been implicated in its pathogenesis (4, 5). Nevertheless, the precise molecular and cellular mechanisms underlying disease onset remain elusive. Increasingly, EMs is recognized as a multifactorial immune-mediated disorder, in which dysregulation of fundamental immunological processes plays a pivotal role in disease initiation and progression (6).

Emerging evidence indicates that nearly all immune cell types in women with EMs exhibit functional abnormalities (7, 8). The disease microenvironment is characterized by aberrant immune cell infiltration, macrophage activation, impaired cytotoxicity of natural killer (NK) cells, and dysregulated expression of proinflammatory and regulatory cytokines (9). Ectopic endometrial cells that survive and proliferate in the peritoneal cavity possess the ability to evade immune surveillance and clearance by resident immune cells, particularly macrophages and NK cells. Mounting evidence now supports a strong association between EMs pathogenesis and impaired NK cell cytotoxicity (10). This review summarizes current progress in understanding the regulatory mechanisms governing NK cell cytotoxicity in EMs, elucidates how ectopic endometrial cells escape NK cell-mediated immune surveillance, and discusses recent advances in NK cell–targeted immunotherapeutic strategies.

## 2 Phenotypes and functions of NK cells

### 2.1 NK cell phenotypes

Natural killer (NK) cells are large granular lymphocytes defined by the CD3^−^CD56^+^CD16^+^/^−^CD57^+^/^−^ immunophenotype. They constitute a central arm of innate immune surveillance, endowed with the capacity to detect and lyse virally infected, malignant, or stressed cells without prior sensitization (11, 12). Beyond their cytolytic role, NK cells can also recognize subsets of normal cells, thereby participating in a broad spectrum of immunological processes, including antigen presentation, regulation of autoimmunity, orchestration of inflammatory responses, modulation of transplant rejection, and maintenance of pregnancy (13). Based on surface expression of CD56 and CD16, NK cells are divided into two major subsets: CD56^dim^CD16^+^ and CD56^bright^CD16^−^ NK cells (14). The CD56^dim^CD16^+^ subset constitutes approximately 90% of circulating NK cells and is highly cytotoxic. In contrast, CD56^bright^CD16^−^ NK cells primarily regulate immune responses via cytokine secretion, such as IFN-γ and TNF-α. Upon appropriate stimulation, CD56^bright^CD16^−^ NK cells can convert into CD56^dim^CD16^+^ NK cells, concomitantly enhancing their cytolytic activity (15). NK cell phenotypic and functional properties are further shaped by their tissue microenvironment. In the endometrium during the menstrual cycle, and in the decidua during pregnancy, NK cells predominantly exhibit the CD56^bright^CD16^−^ phenotype. These cells originate from CD34^+^ progenitors and are involved in spiral artery remodeling, placental development, and maintenance of gestation (16, 17).

### 2.2 NK cell functions

Natural killer (NK) cells exert cytotoxic effects primarily through the exocytosis of cytolytic granules and the induction of apoptosis via Fas ligand (FasL)–mediated signaling (18). Target cell recognition is orchestrated by adhesion molecules in concert with an array of activating and inhibitory receptors, including killer immunoglobulin-like receptors (KIRs), leukocyte immunoglobulin-like receptors (LILRs), and members of the natural killer group 2 (NKG2) receptor family (19). The dynamic equilibrium between these activating and inhibitory cues ultimately dictates the magnitude of NK cell cytotoxicity. In endometriosis (EMs), NK cytotoxic activity has traditionally been evaluated using K562 leukemia cells as targets. Multiple studies have reported diminished lytic capacity of NK cells isolated from both the peripheral blood (20) and peritoneal fluid (21) of patients with EMs. However, because K562 cells lack major histocompatibility complex (MHC) class I molecules, they are intrinsically susceptible to NK-mediated lysis (22), raising concerns about their relevance in modeling EMs-specific immune interactions. A more physiologically relevant approach would be to assess NK cytotoxicity against ectopic endometrial epithelial or stromal cells. Nonetheless, technical challenges, particularly the limited availability of clinical samples and the difficulties in establishing primary cultures, have constrained such investigations. To date, only a small number of studies have employed autologous endometrial cells as targets, and the existing evidence remains insufficient to definitively establish whether NK cell cytotoxicity is reduced within the eutopic endometrium of EMs patients (23).

## 3 Role of NK cells in EMs

### 3.1 NK cell levels in EMs

Most investigations on NK cells in endometriosis (EMs) have examined peripheral blood and peritoneal fluid. Most report no marked differences in the proportions of CD56^+^ and/or CD16^+^ NK cells between EMs patients and healthy controls (24). Some describe reduced CD16^+^CD57^+^ or CD16^+^CD56^−^ subsets (24), whereas others note increased CD56^−^ or CD56^−^CD16^+^ populations (25). Data on NK cells in eutopic versus ectopic endometrial tissue remain scarce. Drury et al. (26) observed that uterine NK (uNK) cell numbers rise from the proliferative to the late secretory phase, peaking before menstruation, in both EMs and non-EMs cases; however, NK cell frequencies were consistently lower in ectopic lesions. Conversely, in women with unexplained recurrent miscarriage or infertility, NKp46^+^/CD56^−^ cells are elevated in the endometrium (27). Furthermore, CD56^−^ or CD16^+^ NK cell counts in ectopic endometrial tissue are generally lower than those in eutopic endometrium of healthy controls. These cells in ectopic lesions also fail to exhibit typical phenotypic and functional profiles seen in uterine NK cells (26).

Recent evidence indicates that distinct NK cell subsets differentially contribute to immune dysregulation in EMs (10). CD56^−^/CD16^+^ NK cells, representing a more differentiated phenotype with potent antibody-dependent cytotoxic potential, are enriched in the peritoneal fluid of EMs patients, yet display functional exhaustion, marked by attenuated degranulation capacity and diminished cytokine release (27–30). Conversely, CD56^+^/CD16^−^ NK cells, typically classified as immature, are relatively expanded in the peripheral circulation and secrete elevated levels of immunoregulatory mediators such as IL-10 and TGF-β, potentially reinforcing local immunosuppression (5, 31). This reciprocal alteration in subset distribution between peripheral blood and peritoneal fluid reflects a phenotypic shift from cytotoxic to immunoregulatory dominance, thereby facilitating lesion persistence and undermining immune surveillance in EMs. Collectively, these findings implicate aberrant NK cell subset composition and functional impairment as central mechanisms driving the loss of NK cytotoxicity in EMs (32).

### 3.2 Role of NK cells in EMs pathogenesis

NK cells are essential components of the innate immune system, forming the first line of defense against pathogens. By eliminating misplaced endometrial cells, they help prevent ectopic implantation. Dysfunctional NK activity or impaired cytotoxicity may contribute to EMs onset. Dorien FO et al. (33) found aberrant expression of NK cell receptors and altered cytokine production by NK cells in the pelvic environment of EMs patients, further implicating their role in disease etiology. He J et al. (34) discovered that sterile alpha motif domain-containing protein 9 (SAMD9) and Ral guanine nucleotide dissociation stimulator-like 2 (RGL2) are significantly upregulated in patients experiencing pelvic pain associated with EMs. Additionally, expression of lysophosphatidic acid receptor 1 (LPAR1) is elevated in ectopic stromal and glandular epithelial cells (34). These findings indicate that NK cells contribute to EMs pathogenesis, particularly in pain phenotypes. Suppression of NK cytotoxic function may exacerbate lesion persistence and pain progression in affected individuals.

### 3.3 Expression of NK cell receptors and ligands in EMs

In endometriosis (EMs), impaired NK cytotoxicity is closely linked to dysregulated activating–inhibitory receptor balance (35). Reduced expression of the activating receptor NKG2D limits NK recognition of ectopic endometrial cells and attenuates perforin/granzyme release, facilitating lesion immune evasion (36). While ULBP-2 levels remain unchanged, the non-classical MHC molecules MICA and MICB are markedly upregulated and correlate with disease severity (37), suggesting potential interference with NK cytotoxicity that warrants further validation. Human leukocyte antigen G (HLA-G), a ligand for inhibitory receptors LILRB1 and KIR2DL4 (38), is aberrantly expressed in both eutopic and ectopic endometrium, with menstrual cycle–dependent variation (39). Elevated soluble HLA-G (sHLA-G) in peritoneal fluid—but not serum—of EMs patients further implicates this axis in NK suppression (39), though its mechanistic role remains unclear (40). Peritoneal NK cells in EMs also exhibit increased expression of the inhibitory receptor NKG2A, which binds HLA-E (41). This interaction dampens degranulation and IFN-γ secretion, paralleling immune escape pathways seen in cancer and supporting lesion persistence (42). Aberrant upregulation of the inhibitory receptor NKG2A on peritoneal NK cells enhances binding to HLA-E on ectopic endometrial cells, amplifying inhibitory signaling and suppressing degranulation, IFN-γ secretion, and overall cytotoxic capacity (32). Adhesion molecule dysregulation further compromises NK function. Effective recognition and stable immunological synapse formation require leukocyte function antigen-1 (LFA-1) on NK cells engaging intercellular adhesion molecule-1 (ICAM-1) on target cells (43, 44). In EMs, ectopic endometrial cells secrete soluble ICAM-1 (sICAM-1), which binds LFA-1 and competitively blocks membrane ICAM-1 interactions, thereby preventing synapse stabilization (45). This disruption reduces perforin/granzyme release and degranulation, weakening cytotoxicity and enabling lesion immune escape. Consistently elevated sICAM-1 levels in peritoneal fluid, together with *in vitro* evidence of NK inhibition (46, 47), identify sICAM-1 as a critical mediator of NK cell dysfunction and lesion survival.

### 3.4 The role of cytokines in regulating NK cell cytotoxicity in EMs patients

**Figure 1:**
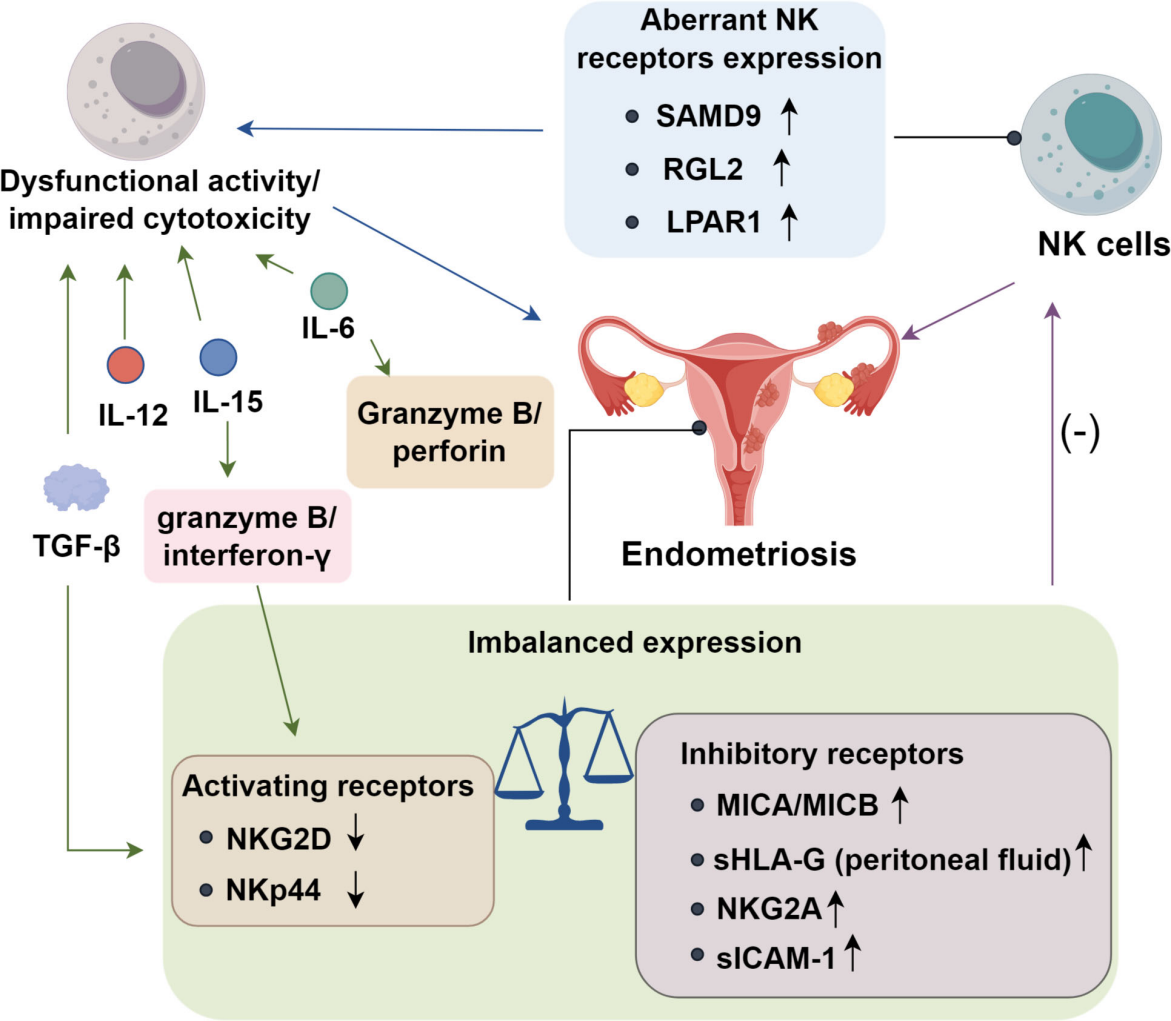
Functions of NK cells in endometriosis progression.

Peritoneal fluid and ectopic endometrial tissue from patients with EMs are enriched in immunosuppressive cytokines, which may disrupt normal immune surveillance (5). Consistent with this milieu, peritoneal fluid, serum, and conditioned supernatants from cultured ectopic endometrium suppress NK-cell cytotoxicity (36, 48). Transforming TGF-β emerges as a central mediator: intraperitoneal TGF-β reduces NK-cell killing and downmodulates the activating receptor NKG2D (36), implicating this pathway in EMs pathogenesis. Additional interleukins further constrain NK function (49). IL-6 signals through JAK/STAT3 to repress transcription of perforin and granzyme B while skewing NK cells toward an anti-inflammatory state with diminished interferon-γ production (48). IL-10, a potent immunosuppressive cytokine, exerts potent immunosuppressive effects by engaging the STAT3/STAT5 signaling axis, while enhancing the expression of inhibitory checkpoints such as NKG2A and PD−1. Collectively, these effects shift NK cells from a cytotoxic to a functionally exhausted phenotype (5). Moreover, elevated IL-12 p40 subunit in the peritoneal fluid may antagonize the activity of the IL-12 heterodimer, thereby impairing NK activation (23). Similarly, IL-15 attenuates NK effector programs by lowering granzyme B and interferon-γ output and reducing expression of stimulatory receptors such as NKG2D and NKp44 (50). Collectively, these cytokine-driven signals establish an immunosuppressive peritoneal niche that dampens NK-cell cytotoxicity and fosters the persistence of ectopic lesions (**Figure 1**).

## 4 NK cell–based immunotherapy for EMs

### 4.1 Cytokine-based strategies

Ectopic endometrial cells in EMs exhibit hallmark features of apoptosis resistance, enhanced adhesion, and invasive capacity, often accompanied by localized angiogenesis during lesion initiation and progression (51). Due to phenotypic similarities with tumor cells, the decline in NK cell cytotoxicity in EMs may represent a form of immune escape, drawing parallels with cancer immune evasion. The modulation of NK cell activity in EMs involves intricate interactions between various activating and inhibitory receptors and their ligands. The downregulation of activating receptors and upregulation of inhibitory ones may be mediated by local immunosuppressive cytokines. Therefore, targeting these inhibitory factors represents a potential strategy to restore NK cell cytotoxicity.

IL-2 is a prototypical NK-stimulatory cytokine capable of reversing NK cell suppression. IL-2 stimulation leads to the generation of lymphokine-activated killer (LAK) cells, which exhibit high cytotoxicity against drug-resistant tumor cells, suggesting potential application in cancer immunotherapy (52). Notably, LAK cells have demonstrated cytotoxicity toward various target cells, including endometrial cells from EMs patients (53). In rat models of EMs, IL-2 administration enhances intrauterine immune activation and leads to failure of ectopic implantation (22). Cytokine combinations based on IL−15 and IL−2 have garnered attention. IL-15 sustains NK cell proliferation and survival without expanding regulatory T cells that are typically induced by IL-2 (54, 55). IL-21 synergizes with IL-15 to further augment NK cytotoxicity and cytokine secretion. Preclinical studies in various immune-mediated disorders indicate that such cytokine combinations can markedly enhance NK effector functions (56, 57), suggesting their potential translational value in EMs. These findings suggest that cytokine stimulation therapy may offer a viable avenue for immunotherapy in EMs.

### 4.2 Programmed death-1/programmed death ligand-1 pathway

The programmed death-1 (PD-1) and programmed death-ligand 1 (PD-L1) checkpoint axis is a prominent focus in NK cell–based immunotherapy (58). PD-1 and PD-L1 expression have been detected in ectopic endometrial tissues (59, 60). In cancer immunotherapy, monoclonal antibodies targeting PD-1 and PD-L1 have yielded promising outcomes. However, such interventions may also trigger extensive adverse effects across multiple tissues and organs (61). Nonetheless, targeting the PD-1/PD-L1 axis remains a promising immunotherapeutic direction for EMs. Elevated expression of the inhibitory receptor NKG2A (which recognizes HLA-E molecules) has been observed in Ems (55). HLA-E is commonly expressed in various tumor types, and clinical trials have shown favorable responses to anti-NKG2A antibodies in certain cancers (62). Although the functional role of NKG2A in EMs remains to be fully elucidated, other checkpoint pathways, including those mediated by KIR2DL1 (binding HLA-C2) and LILRB1 (binding HLA-G), may also serve as potential immunotherapeutic targets. Importantly, the balance between activating and inhibitory signals is essential for optimal NK cell function. Excessive NK activation could risk collateral tissue damage, underscoring the necessity for cautious selection and precise application of NK-based immunotherapy for EMs.

### 4.3 Adoptive NK cell transfer and CAR−NK therapy

Adoptive transfer of NK cells seeks to reconstitute cytotoxic activity within the peritoneal cavity and can be executed with autologous, haploidentical, cord-blood, peripheral-blood, or induced pluripotent stem cell (iPSC)–derived NK products (63). Moreover, the development of chimeric antigen receptor NK cells (CAR-NK) allows for redirection of NK cells against specific targets (64). Recent CAR-NK designs frequently incorporate “armoring” with membrane-bound or secreted IL-15 to improve *in-vivo* persistence and metabolic fitness; genome editing to remove intracellular checkpoints such as CISH further augments IL-15 signaling and antitumor function (65). iPSC-derived NK platforms also introduce a high-affinity, non-cleavable CD16 (hnCD16) to sustain ADCC and enable combination with tumor-targeting antibodies (66). To enhance homing to diseased tissues, NK or CAR-NK cells can be retargeted with chemokine receptors (CXCR1/CXCR4), which improves trafficking in preclinical models (67–69). Collectively, these modifications address the historical challenges of NK persistence, trafficking, and serial killing in solid-tissue settings (70, 71).

The first-in-human, cord-blood–derived anti-CD19/IL-15 CAR-NK trial demonstrated rapid responses in 8/11 patients (73%) with minimal CRS/neurotoxicity and detectable persistence up to 12 months (72). An iPSC-derived CAR-NK product (FT596; includes CD19 CAR, IL-15 receptor fusion, and hnCD16) showed tolerability and objective responses in a Phase 1 study, supporting feasibility of standardized “off-the-shelf” CAR-NK therapy (73). Additional early-phase programs (NKG2D-ligand–targeted and CD19-targeted allogeneic CAR-NK) are progressing with preliminary activity and acceptable safety in Phase 1 settings (74). At present, clinical trial testing CAR-NK specifically for EMs is limited. However, studies highlight adoptive NK-based approaches under evaluation for severe EMs, and preclinical data support that exogenous NK cells can infiltrate peritoneal/ovarian lesions and may be delivered via routes including intraperitoneal administration (55, 75, 76). Key hurdles include identifying lesion-restricted antigens to avoid off-target cytotoxicity, improving trafficking and retention within ectopic implants (chemokine-receptor retargeting), and mitigating the immunosuppressive peritoneal milieu (TGF-β, IL-6, IL-10), potentially via IL-15 armoring or combination checkpoint blockade (77). Given the accumulating safety data and modular engineering options (78–80), CAR-NK strategies merit staged translation in EMs once lesion-specific targets and homing cues are defined.

## 5 Conclusion

In summary, NK cell dysfunction is a central immune defect in endometriosis, driven by microenvironmental immunosuppression and ectopic cell immune evasion through altered receptor-ligand interactions, adhesion molecule aberrations, and cytokine-mediated suppression, which collectively impair cytotoxic clearance of ectopic lesions. While emerging immunotherapies targeting NK cells—such as checkpoint blockade, cytokine stimulation, and adoptive cell therapy—hold translational potential, challenges remain in optimizing specificity and safety to avoid systemic autoimmunity.

However, several obstacles need to be addressed before NK cell–based immunotherapy can be widely applied in EMs. Antigenic heterogeneity of ectopic lesions complicates the identification of reliable NK cell targets; the limited trafficking and retention of NK cells within peritoneal and pelvic lesions may reduce therapeutic efficacy; and systemic activation of NK cells carries a risk of off-target cytotoxicity and tissue damage. These considerations highlight the importance of carefully designed, patient-tailored approaches and combination strategies. Future research must prioritize human studies, biomarker-driven patient stratification, and combinatorial approaches integrating NK-targeted agents with existing hormonal or surgical therapies to improve clinical outcomes for pain and infertility in EMs.

## References

[1] Ellis K Munro D Clarke J. Endometriosis is undervalued: A call to action. Front Glob Womens Health. (2022) 3:902371. doi: 10.3389/fgwh.2022.902371, PMID: 35620300 PMC9127440

[2] Parasar P Ozcan P Terry KL. Endometriosis: epidemiology, diagnosis and clinical management. Curr Obstet Gynecol Rep. (2017) 6:34–41. doi: 10.1007/s13669-017-0187-1, PMID: 29276652 PMC5737931

[3] Leone Roberti Maggiore U Chiappa V Ceccaroni M Roviglione G Savelli L Ferrero S. Epidemiology of infertility in women with endometriosis. Best Pract Res Clin Obstet Gynaecol. (2024) 92:102454. doi: 10.1016/j.bpobgyn.2023.102454, PMID: 38183767

[4] Zhou Y Li J Chen M Huang H. Identification and validation of immune-related and inflammation-related genes in endometriosis. Front Endocrinol (Lausanne). (2025) 16:1545670. doi: 10.3389/fendo.2025.1545670, PMID: 40405976 PMC12095003

[5] Zhou WJ Yang HL Shao J Mei J Chang KK Zhu R. Anti-inflammatory cytokines in endometriosis. Cell Mol Life Sci. (2019) 76:2111–32. doi: 10.1007/s00018-019-03056-x, PMID: 30826860 PMC11105498

[6] Ochoa Bernal MA Fazleabas AT. The known, the unknown and the future of the pathophysiology of endometriosis. Int J Mol Sci. (2024) 25:5815. doi: 10.3390/ijms25115815, PMID: 38892003 PMC11172035

[7] Chen S Liu Y Zhong Z Wei C Liu Y Zhu X. Peritoneal immune microenvironment of endometriosis: Role and therapeutic perspectives. Front Immunol. (2023) 14:1134663. doi: 10.3389/fimmu.2023.1134663, PMID: 36865552 PMC9971222

[8] Pan L Chen Y Zhou Z Ma S Cao Y Ma Y. The correlation between immune cells and endometriosis: a bidirectional two-sample mendelian randomization study. BMC Womens Health. (2024) 24:641. doi: 10.1186/s12905-024-03493-2, PMID: 39702192 PMC11660437

[9] Symons LK Miller JE Kay VR Marks RM Liblik K Koti M. The immunopathophysiology of endometriosis. Trends Mol Med. (2018) 24:748–62. doi: 10.1016/j.molmed.2018.07.004, PMID: 30054239

[10] Reis JL Rosa NN Martins C Ângelo-Dias M Borrego LM Lima J. The role of NK and T cells in endometriosis. Int J Mol Sci. (2024) 25:10141. doi: 10.3390/ijms251810141, PMID: 39337624 PMC11432446

[11] Nielsen CM White MJ Goodier MR Riley EM. Functional significance of CD57 expression on human NK cells and relevance to disease. Front Immunol. (2013) 4:422. doi: 10.3389/fimmu.2013.00422, PMID: 24367364 PMC3856678

[12] Brauning A Rae M Zhu G Fulton E Admasu TD Stolzing A. Aging of the immune system: focus on natural killer cells phenotype and functions. Cells. (2022) 11:1017. doi: 10.3390/cells11061017, PMID: 35326467 PMC8947539

[13] Ścieżyńska A Komorowski M Soszyńska M Malejczyk J. NK cells as potential targets for immunotherapy in endometriosis. J Clin Med. (2019) 8:1468. doi: 10.3390/jcm8091468, PMID: 31540116 PMC6780982

[14] Forconi CS Oduor CI Oluoch PO Ong’echa JM Münz C Bailey JA. A new hope for CD56(neg)CD16(pos) NK cells as unconventional cytotoxic mediators: an adaptation to chronic diseases. Front Cell Infect Microbiol. (2020) 10:162. doi: 10.3389/fcimb.2020.00162, PMID: 32373555 PMC7186373

[15] Di Vito C Mikulak J Mavilio D. On the way to become a natural killer cell. Front Immunol. (2019) 10:1812. doi: 10.3389/fimmu.2019.01812, PMID: 31428098 PMC6688484

[16] Bulmer JN Lash GE. Uterine natural killer cells: Time for a re-appraisal? F1000Res. (2019) 8:999. doi: 10.12688/f1000research, PMID: 31316752 PMC6611138

[17] Xie M Li Y Meng YZ Xu P Yang YG Dong S. Uterine natural killer cells: A rising star in human pregnancy regulation. Front Immunol. (2022) 13:918550. doi: 10.3389/fimmu.2022.918550, PMID: 35720413 PMC9198966

[18] Oshimi Y Oda S Honda Y Nagata S Miyazaki S. Involvement of Fas ligand and Fas-mediated pathway in the cytotoxicity of human natural killer cells. J Immunol. (1996) 157:2909–15. doi: 10.4049/jimmunol.157.7.2909, PMID: 8816396

[19] Rajalingam R. Diversity of killer cell immunoglobulin-like receptors and disease. Clin Lab Med. (2018) 38:637–53. doi: 10.1016/j.cll.2018.08.001, PMID: 30420058

[20] Quaranta MG Porpora MG Mattioli B Giordani L Libri I Ingelido AM. Impaired NK-cell-mediated cytotoxic activity and cytokine production in patients with endometriosis: a possible role for PCBs and DDE. Life Sci. (2006) 79:491–8. doi: 10.1016/j.lfs.2006.01.026, PMID: 16499933

[21] Jeung IC Chung YJ Chae B Kang SY Song JY Jo HH. Effect of helixor A on natural killer cell activity in endometriosis. Int J Med Sci. (2015) 12:42–7. doi: 10.7150/ijms.10076, PMID: 25552917 PMC4278874

[22] Wang X Cabrera FG Sharp KL Spencer DM Foster AE Bayle JH. Engineering tolerance toward allogeneic CAR-T cells by regulation of MHC surface expression with human herpes virus-8 proteins. Mol Ther. (2021) 29:718–33. doi: 10.1016/j.ymthe.2020.10.019, PMID: 33554868 PMC7854355

[23] Mazzeo D Viganó P Di Blasio AM Sinigaglia F Vignali M Panina-Bordignon P. Interleukin-12 and its free p40 subunit regulate immune recognition of endometrial cells: potential role in endometriosis. J Clin Endocrinol Metab. (1998) 83:911–6. doi: 10.1210/jc.83.3.911, PMID: 9506747

[24] Szyllo K Tchorzewski H Banasik M Glowacka E Lewkowicz P Kamer-Bartosinska A. The involvement of T lymphocytes in the pathogenesis of endometriotic tissues overgrowth in women with endometriosis. Mediators Inflammation. (2003) 12:131–8. doi: 10.1080/0962935031000134842, PMID: 12857596 PMC1781609

[25] Dias JA Jr. Podgaec S de Oliveira RM Carnevale Marin ML Baracat EC Abrão MS. Patients with endometriosis of the rectosigmoid have a higher percentage of natural killer cells in peripheral blood. J Minim Invasive Gynecol. (2012) 19:317–24. doi: 10.1016/j.jmig.2011.12.021, PMID: 22348900

[26] Drury JA Parkin KL Coyne L Giuliani E Fazleabas AT Hapangama DK. The dynamic changes in the number of uterine natural killer cells are specific to the eutopic but not to the ectopic endometrium in women and in a baboon model of endometriosis. Reprod Biol Endocrinol. (2018) 16:67. doi: 10.1186/s12958-018-0385-3, PMID: 30021652 PMC6052567

[27] Giuliani E Parkin KL Lessey BA Young SL Fazleabas AT. Characterization of uterine NK cells in women with infertility or recurrent pregnancy loss and associated endometriosis. Am J Reprod Immunol. (2014) 72:262–9. doi: 10.1111/aji.12259, PMID: 24807109 PMC4126872

[28] Cocker ATH Guethlein LA Parham P. The CD56-CD16+ NK cell subset in chronic infections. Biochem Soc Trans. (2023) 51:1201–12. doi: 10.1042/BST20221374, PMID: 37140380

[29] Hosseinzadeh R Moini A Hosseini R Fatehnejad M Yekaninejad MS Javidan M. A higher number of exhausted local PD1+, but not TIM3+, NK cells in advanced endometriosis. Heliyon. (2024) 10:e23294. doi: 10.1016/j.heliyon.2023.e23294, PMID: 38173487 PMC10761348

[30] Kang YJ Jeung IC Park A Park YJ Jung H Kim TD. An increased level of IL-6 suppresses NK cell activity in peritoneal fluid of patients with endometriosis via regulation of SHP-2 expression. Hum Reprod. (2014) 29:2176–89. doi: 10.1093/humrep/deu172, PMID: 25035432

[31] Yang S Wang H Li D Li M. An estrogen-NK cells regulatory axis in endometriosis, related infertility, and miscarriage. Int J Mol Sci. (2024) 25:3362. doi: 10.3390/ijms25063362, PMID: 38542336 PMC10970045

[32] Reis JL Rosa NN Ângelo-Dias M Martins C Borrego LM Lima J. Natural killer cell receptors and endometriosis: A systematic review. Int J Mol Sci. (2022) 24:331. doi: 10.3390/ijms24010331, PMID: 36613776 PMC9820702

[33] DF O Roskams T Van den Eynde K Vanhie A Peterse DP Meuleman C. The presence of endometrial cells in peritoneal fluid of women with and without endometriosis. Reprod Sci. (2017) 24:242–51. doi: 10.1177/1933719116653677, PMID: 27324432

[34] He J Xu Y Yi M Gu C Zhu Y Hu G. Involvement of natural killer cells in the pathogenesis of endometriosis in patients with pelvic pain. J Int Med Res. (2020) 48:300060519871407. doi: 10.1177/0300060519871407, PMID: 32727237 PMC7394034

[35] Björk E Israelsson P Nagaev I Nagaeva O Lundin E Ottander U. Endometriotic tissue-derived exosomes downregulate NKG2D-mediated cytotoxicity and promote apoptosis: mechanisms for survival of ectopic endometrial tissue in endometriosis. J Immunol. (2024) 213:567–76. doi: 10.4049/jimmunol.2300781, PMID: 38984872 PMC11335327

[36] Liu ZQ Lu MY Liu B. Circulating CD56+ NKG2D+ NK cells and postoperative fertility in ovarian endometrioma. Sci Rep. (2020) 10:18598. doi: 10.1038/s41598-020-75570-z, PMID: 33122818 PMC7596045

[37] González-Foruria I Santulli P Chouzenoux S Carmona F Batteux F Chapron C. Soluble ligands for the NKG2D receptor are released during endometriosis and correlate with disease severity. PloS One. (2015) 10:e0119961. doi: 10.1371/journal.pone.0119961, PMID: 25775242 PMC4361401

[38] Attia JVD Dessens CE van de Water R Houvast RD Kuppen PJK Krijgsman D. The molecular and functional characteristics of HLA-G and the interaction with its receptors: where to intervene for cancer immunotherapy? Int J Mol Sci. (2020) 21:8678. doi: 10.3390/ijms21228678, PMID: 33213057 PMC7698525

[39] Rached MR Coelho V Marin MLC Pincerato K Fujita A Kalil JE. HLA-G is upregulated in advanced endometriosis. Eur J Obstet Gynecol Reprod Biol. (2019) 235:36–41. doi: 10.1016/j.ejogrb.2019.01.030, PMID: 30784825

[40] Santoso B Sa’adi A Dwiningsih SR Tunjungseto A Widyanugraha MYA Mufid AF. Soluble immune checkpoints CTLA-4, HLA-G, PD-1, and PD-L1 are associated with endometriosis-related infertility. Am J Reprod Immunol. (2020) 84:e13296. doi: 10.1111/aji.13296, PMID: 32593225

[41] Bartel Y Bauer B Steinle A. Modulation of NK cell function by genetically coupled C-type lectin-like receptor/ligand pairs encoded in the human natural killer gene complex. Front Immunol. (2013) 4:362. doi: 10.3389/fimmu.2013.00362, PMID: 24223577 PMC3819593

[42] Galandrini R Porpora MG Stoppacciaro A Micucci F Capuano C Tassi I. Increased frequency of human leukocyte antigen-E inhibitory receptor CD94/NKG2A-expressing peritoneal natural killer cells in patients with endometriosis. Fertil Steril. (2008) 89:1490–6. doi: 10.1016/j.fertnstert.2007.05.018, PMID: 17706207

[43] Eitler J Rackwitz W Wotschel N Gudipati V Murali Shankar N Sidorenkova A. CAR-mediated targeting of NK cells overcomes tumor immune escape caused by ICAM-1 downregulation. J Immunother Cancer. (2024) 12:e008155. doi: 10.1136/jitc-2023-008155, PMID: 38417916 PMC10900364

[44] Pariani AP Almada E Hidalgo F Borini-Etichetti C Vena R Marín L. Identification of a novel mechanism for LFA-1 organization during NK cytolytic response. J Cell Physiol. (2023) 238:227–41. doi: 10.1002/jcp.30921, PMID: 36477412

[45] Viganó P Pardi R Magri B Busacca M Di Blasio AM Vignali M. Expression of intercellular adhesion molecule-1 (ICAM-1) on cultured human endometrial stromal cells and its role in the interaction with natural killers. Am J Reprod Immunol. (1994) 32:139–45. doi: 10.1111/j.1600-0897.1994.tb01104.x, PMID: 7880394

[46] Somigliana E Viganò P Gaffuri B Guarneri D Busacca M Vignali M. Human endometrial stromal cells as a source of soluble intercellular adhesion molecule (ICAM)-1 molecules. Hum Reprod. (1996) 11:1190–4. doi: 10.1093/oxfordjournals.humrep.a019353, PMID: 8671421

[47] Fukaya T Sugawara J Yoshida H Murakami T Yajima A. Intercellular adhesion molecule-1 and hepatocyte growth factor in human endometriosis: original investigation and a review of literature. Gynecol Obstet Invest. (1999) 47 Suppl 1:11–16; discussion 16-17. doi: 10.1159/000052854, PMID: 10087423

[48] Prins JR Marissen LM Scherjon SA Hoek A Cantineau AEP. Is there an immune modulating role for follicular fluid in endometriosis? A narrative review. Reproduction. (2020) 159:R45–r54. doi: 10.1530/REP-19-0050, PMID: 31370001

[49] Jeung I Cheon K Kim MR. Decreased cytotoxicity of peripheral and peritoneal natural killer cell in endometriosis. BioMed Res Int. (2016) 2016:2916070. doi: 10.1155/2016/2916070, PMID: 27294113 PMC4880704

[50] Bellelis P Frediani Barbeiro D Gueuvoghlanian-Silva BY Kalil J Abrão MS Podgaec S. Interleukin-15 and interleukin-7 are the major cytokines to maintain endometriosis. Gynecol Obstet Invest. (2019) 84:435–44. doi: 10.1159/000496607, PMID: 30712043

[51] Dinsdale N Nepomnaschy P Crespi B. The evolutionary biology of endometriosis. Evol Med Public Health. (2021) 9:174–91. doi: 10.1093/emph/eoab008, PMID: 33854783 PMC8030264

[52] López-Díaz de Cerio A García-Muñoz R Pena E Panizo Á Feliu J Giraldo P. Maintenance therapy with ex vivo expanded lymphokine-activated killer cells and rituximab in patients with follicular lymphoma is safe and may delay disease progression. Br J Haematol. (2020) 189:1064–73. doi: 10.1111/bjh.16474, PMID: 32130737

[53] Velasco I Quereda F Bermejo R Campos A Acién P. Intraperitoneal recombinant interleukin-2 activates leukocytes in rat endometriosis. J Reprod Immunol. (2007) 74:124–32. doi: 10.1016/j.jri.2006.12.001, PMID: 17210185

[54] Yang Y Lundqvist A. Immunomodulatory effects of IL-2 and IL-15; implications for cancer immunotherapy. Cancers (Basel). (2020) 12:3586. doi: 10.3390/cancers12123586, PMID: 33266177 PMC7761238

[55] Hoogstad-van Evert J Paap R Nap A van der Molen R. The promises of natural killer cell therapy in endometriosis. Int J Mol Sci. (2022) 23:5539. doi: 10.3390/ijms23105539, PMID: 35628346 PMC9146217

[56] Wagner J Pfannenstiel V Waldmann A Bergs JWJ Brill B Huenecke S. A two-phase expansion protocol combining interleukin (IL)-15 and IL-21 improves natural killer cell proliferation and cytotoxicity against rhabdomyosarcoma. Front Immunol. (2017) 8:676. doi: 10.3389/fimmu.2017.00676, PMID: 28659917 PMC5466991

[57] Heinze A Grebe B Bremm M Huenecke S Munir TA Graafen L. The Synergistic Use of IL-15 and IL-21 for the Generation of NK Cells From CD3/CD19-Depleted Grafts Improves Their ex vivo Expansion and Cytotoxic Potential Against Neuroblastoma: Perspective for Optimized Immunotherapy Post Haploidentical Stem Cell Transplantation. Front Immunol. (2019) 10:2816. doi: 10.3389/fimmu.2019.02816, PMID: 31849984 PMC6901699

[58] Hsu J Hodgins JJ Marathe M Nicolai CJ Bourgeois-Daigneault MC Trevino TN. Contribution of NK cells to immunotherapy mediated by PD-1/PD-L1 blockade. J Clin Invest. (2018) 128:4654–68. doi: 10.1172/JCI99317, PMID: 30198904 PMC6159991

[59] Suszczyk D Skiba W Zardzewiały W Pawłowska A Włodarczyk K Polak G. Clinical value of the PD-1/PD-L1/PD-L2 pathway in patients suffering from endometriosis. Int J Mol Sci. (2022) 23:11607. doi: 10.3390/ijms231911607, PMID: 36232911 PMC9570092

[60] Suszczyk D Skiba W Pawłowska-Łachut A Dymanowska-Dyjak I Włodarczyk K Paduch R. Immune checkpoints in endometriosis-A new insight in the pathogenesis. Int J Mol Sci. (2024) 25:6266. doi: 10.3390/ijms25116266, PMID: 38892453 PMC11172867

[61] Sosa A Lopez Cadena E Simon Olive C Karachaliou N Rosell R. Clinical assessment of immune-related adverse events. Ther Adv Med Oncol. (2018) 10:1758835918764628. doi: 10.1177/1758835918764628, PMID: 29623110 PMC5882039

[62] Sivori S Vacca P Del Zotto G Munari E Mingari MC Moretta L. Human NK cells: surface receptors, inhibitory checkpoints, and translational applications. Cell Mol Immunol. (2019) 16:430–41. doi: 10.1038/s41423-019-0206-4, PMID: 30778167 PMC6474200

[63] Lin X Sun Y Dong X Liu Z Sugimura R Xie G. IPSC-derived CAR-NK cells for cancer immunotherapy. BioMed Pharmacother. (2023) 165:115123. doi: 10.1016/j.biopha.2023.115123, PMID: 37406511

[64] Valeri A García-Ortiz A Castellano E Córdoba L Maroto-Martín E Encinas J. Overcoming tumor resistance mechanisms in CAR-NK cell therapy. Front Immunol. (2022) 13:953849. doi: 10.3389/fimmu.2022.953849, PMID: 35990652 PMC9381932

[65] Daher M Basar R Gokdemir E Baran N Uprety N Nunez Cortes AK. Targeting a cytokine checkpoint enhances the fitness of armored cord blood CAR-NK cells. Blood. (2021) 137:624–36. doi: 10.1182/blood.2020007748, PMID: 32902645 PMC7869185

[66] Zhu H Blum RH Bjordahl R Gaidarova S Rogers P Lee TT. Pluripotent stem cell-derived NK cells with high-affinity noncleavable CD16a mediate improved antitumor activity. Blood. (2020) 135:399–410. doi: 10.1182/blood.2019000621, PMID: 31856277 PMC7005364

[67] Ng YY Tay JCK Wang S. CXCR1 expression to improve anti-cancer efficacy of intravenously injected CAR-NK cells in mice with peritoneal xenografts. Mol Ther Oncolytics. (2020) 16:75–85. doi: 10.1016/j.omto.2019.12.006, PMID: 31970285 PMC6965500

[68] Levy E Reger R Segerberg F Lambert M Leijonhufvud C Baumer Y. Enhanced bone marrow homing of natural killer cells following mRNA transfection with gain-of-function variant CXCR4(R334X). Front Immunol. (2019) 10:1262. doi: 10.3389/fimmu.2019.01262, PMID: 31231387 PMC6560173

[69] Yoon JH Yoon HN Kang HJ Yoo H Choi MJ Chung JY. Empowering pancreatic tumor homing with augmented anti-tumor potency of CXCR2-tethered CAR-NK cells. Mol Ther Oncol. (2024) 32:200777. doi: 10.1016/j.omton.2024.200777, PMID: 38596297 PMC10926211

[70] Chu J Gao F Yan M Zhao S Yan Z Shi B. Natural killer cells: a promising immunotherapy for cancer. J Transl Med. (2022) 20:240. doi: 10.1186/s12967-022-03437-0, PMID: 35606854 PMC9125849

[71] Page A Chuvin N Valladeau-Guilemond J Depil S. Development of NK cell-based cancer immunotherapies through receptor engineering. Cell Mol Immunol. (2024) 21:315–31. doi: 10.1038/s41423-024-01145-x, PMID: 38443448 PMC10978891

[72] Liu E Marin D Banerjee P Macapinlac HA Thompson P Basar R. Use of CAR-transduced natural killer cells in CD19-positive lymphoid tumors. N Engl J Med. (2020) 382:545–53. doi: 10.1056/NEJMoa1910607, PMID: 32023374 PMC7101242

[73] Ghobadi A Bachanova V Patel K Park JH Flinn I Riedell PA. Induced pluripotent stem-cell-derived CD19-directed chimeric antigen receptor natural killer cells in B-cell lymphoma: a phase 1, first-in-human trial. Lancet. (2025) 405:127–36. doi: 10.1016/S0140-6736(24)02462-0, PMID: 39798981 PMC11827677

[74] Jørgensen LV Christensen EB Barnkob MB Barington T. The clinical landscape of CAR NK cells. Exp Hematol Oncol. (2025) 14:46. doi: 10.1186/s40164-025-00633-8, PMID: 40149002 PMC11951618

[75] Dai Y Ye Z Lin X Zhang S. Immunopathological insights into endometriosis: from research advances to future treatments. Semin Immunopathol. (2025) 47:31. doi: 10.1007/s00281-025-01058-5, PMID: 40679648 PMC12274256

[76] Montenegro ML Ferriani RA Basse PH. Exogenous activated NK cells enhance trafficking of endogenous NK cells to endometriotic lesions. BMC Immunol. (2015) 16:51. doi: 10.1186/s12865-015-0105-0, PMID: 26318622 PMC4552996

[77] Balkhi S Zuccolotto G Di Spirito A Rosato A Mortara L. CAR-NK cell therapy: promise and challenges in solid tumors. Front Immunol. (2025) 16:1574742. doi: 10.3389/fimmu.2025.1574742, PMID: 40260240 PMC12009813

[78] Zhu X Xue J Jiang H Xue D. CAR-NK cells for gastrointestinal cancer immunotherapy: from bench to bedside. Mol Cancer. (2024) 23:237. doi: 10.1186/s12943-024-02151-3, PMID: 39443938 PMC11515662

[79] Marin D Li Y Basar R Rafei H Daher M Dou J. Safety, efficacy and determinants of response of allogeneic CD19-specific CAR-NK cells in CD19(+) B cell tumors: a phase 1/2 trial. Nat Med. (2024) 30:772–84. doi: 10.1038/s41591-023-02785-8, PMID: 38238616 PMC10957466

[80] Lei W Liu H Deng W Chen W Liang Y Gao W. Safety and feasibility of 4-1BB co-stimulated CD19-specific CAR-NK cell therapy in refractory/relapsed large B cell lymphoma: a phase 1 trial. Nat Cancer. (2025) 6:786–800. doi: 10.1038/s43018-025-00940-3, PMID: 40251398 PMC12122374

